# Systems Pharmacology-Based Approach to Comparatively Study the Independent and Synergistic Mechanisms of Danhong Injection and Naoxintong Capsule in Ischemic Stroke Treatment

**DOI:** 10.1155/2019/1056708

**Published:** 2019-02-04

**Authors:** Junfeng Zhu, Xiaojiao Yi, Yiwen Zhang, Zongfu Pan, Like Zhong, Ping Huang

**Affiliations:** ^1^Laboratory of Clinical Pharmacy, Zhejiang Cancer Hospital, Hangzhou 310022, China; ^2^College of Pharmaceutical Sciences, Zhejiang University, Hangzhou 310058, China

## Abstract

To provide evidence for the better clinical use of traditional Chinese medicine preparations (TCMPs), comparison of the pharmacological mechanisms between TCMPs with similar therapeutic effect is necessary. However, methodology for dealing with this issue is still scarce. Danhong injection (DHI) and Naoxintong capsule (NXT) are representative TCMPs for ischemic stroke (IS) treatment, which are also frequently used in combination. Here they were employed as research objects to demonstrate the feasibility of systems pharmacology approach in elucidation of the independent and combined effect of TCMPs. By incorporating chemical screening, target prediction, and network construction, a feasible systems pharmacology model has been established to systematically uncover the underlying action mechanisms of DHI, NXT, or their pair in IS treatment. Systematic analysis of the created TCMP-Compound-Target-Disease network revealed that DHI and NXT shared common targets such as PTGS2, F2, ADRB1, IL6, ALDH2, and CCL2, which were involved in the vasomotor system regulation, blood-brain barrier disruption, redox imbalance, neurotrophin activity, and brain inflammation. In comparative mechanism study, the merged DHI/NXT-IS PPI network and pathway enrichment analysis indicated that DHI and NXT exerted the therapeutic effects mainly through immune system and VEGF signaling pathways. Meanwhile, they had their own unique pathways, e.g., calcium signaling pathway for DHI and gap junction for NXT. While for their synergistic mechanism, DHI and NXT participated in chemokine signaling pathway, T cell receptor signaling pathway, VEGF signaling pathway, gap junction, and so on. Our study provided an optimized strategy for dissecting the different and combined effect of TCMPs with similar actions.

## 1. Introduction

Ischemic stroke (IS) is a common disease caused by intracranial or neck blood vessel occlusion that impairs blood flow to a portion of the brain and leads to subsequent cerebral necrosis [1]. Due to its high morbidity and mortality, as well as high-risk of permanent disability in surviving individuals, IS represents a substantial economic and social burden throughout the world [2, 3]. Currently, pharmacological or mechanical thrombolytic therapy for restoration of blood flow to ischemic tissue is the main treatment to improve patient outcome [4, 5]. However, their unique benefits are shadowed by limited therapeutic time window and serious complications [6]. Moreover, cellular consequences of ischemic brain injury are very complex and chemical drugs are hardly to achieve favorable outcomes because of their single therapeutic target [7–9]. Thus, novel therapeutic drugs and strategies are urgently needed to improve the efficiency in treating IS.

Traditional Chinese medicine (TCM), characterized as multiple compounds and targets, differs in substance, methodology, and philosophy to chemical drugs [10]. It has been practiced in China for thousands of years and is now becoming more frequently used in the West. In recent years, TCM preparations (TCMPs) have been proposed as important complementary and alternative medicines for IS treatment [11, 12]. Danhong injection (DHI) and Naoxintong capsule (NXT) are both commonly used TCMPs for the prevention and treatment of cardiovascular and cerebrovascular diseases [13–16]. DHI is extracted from Radix Salviae Miltiorrhizae and Flos Carthami, while NXT consists of 16 kinds of herbs including Radix Astragali, Radix Paeoniae Rubra, Radix Salviae Miltiorrhizae, and so on. Their efficiency in protecting against IS has been widely validated, whether by used alone or in combination [17–21]. According to a recent report, DHI-NXT combination therapy could achieve better therapeutic effect than monotherapy in cerebral ischemia-reperfusion model of rats [22]. However, the comparative and synergistic effect of the action mechanisms between DHI and NXT still remain vague and warrant further investigation.

As the multicomponent and multitarget nature of TCMPs, it is very difficult to systematically study their mechanisms using routine methods [23]. Therefore, systems pharmacology method is now widely perceived as an integral and efficient tool to study the complex molecular mechanisms of herbal medicines [24–30]. This approach usually includes three steps, including active compounds screening, drug targets prediction, and network/pathway analysis [31, 32]. It could provide global active compounds-therapeutic targets interactions based on the network pharmacology framework, which matches well with the integrity and systemic conception of TCM. For example, a network of 31 active compounds interacting with 42 disease targets was constructed by Yue et al. to elucidate the polypharmacological mechanisms underlying the efficiency of herb pair Danggui-Honghua for blood stasis syndrome treatment [33]. Still, methodology for comparative study of the mechanisms between TCMPs is scarce.

To provide evidence for the better clinical use of DHI and NXT, here we proposed a feasible systems pharmacology-based method to explore the similarities and differences of the pharmacological mechanisms between DHI and NXT. Their synergistic mechanism in treatment of IS was also investigated. To our knowledge, no work has been reported about the mechanism comparison and synergistic effect of DHI and NXT.

## 2. Results and Discussion

**Figure 1:**
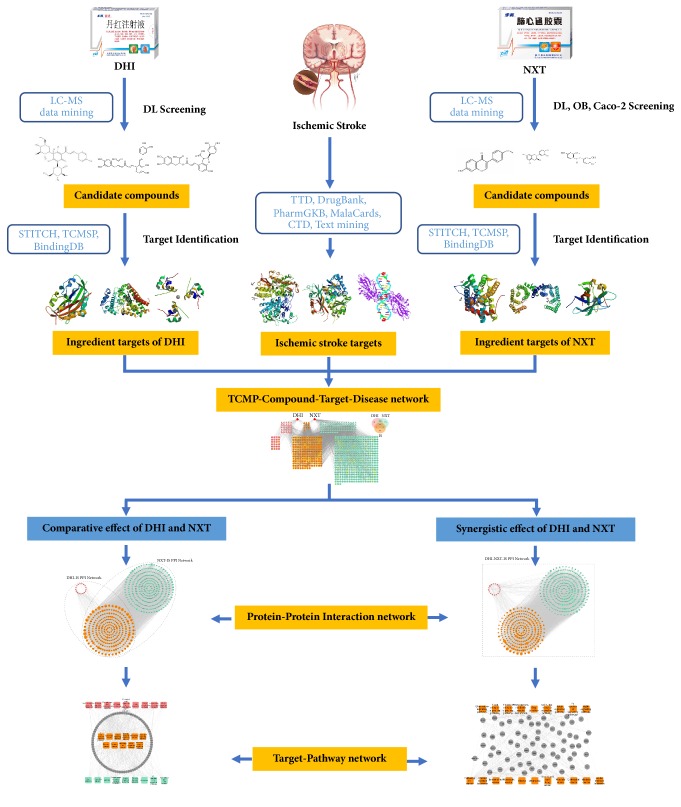
Workflow for dissecting the comparative and synergistic effects of DHI and NXT against IS.

In this study, a network pharmacology approach based on chemical, pharmacokinetic, and pharmacological data was employed to systematically investigate the comparative and synergistic effects of DHI and NXT against IS. The overall procedure involved five steps (Figure 1): (1) the chemical ingredients of DHI and NXT were collected and evaluated by* in silico* ADME (absorption, distribution, metabolism, and excretion) system to screen candidate compounds; (2) the putative targets of the candidate compounds and IS were data-mined from various databases and literatures; (3) a basal TCMP-Compound-Target-Disease network was constructed to illustrate the interaction among DHI/NXT, ingredients, targets and IS; (4) for comparative study of the action mechanisms, two protein-protein interaction (PPI) networks named DHI-IS and NXT-IS PPI network were merged and pathway enrichment analysis was separately performed to explore the common and unique mechanisms of DHI and NXT; (5) for the study of their synergistic effect, DHI-NXT-IS PPI network was established among DHI-ingredient targets, NXT-ingredient targets, and IS targets. The enriched pathways were analyzed to explain the synergistic mechanisms of the drug combination.

### 2.1. Candidate Ingredients in DHI and NXT

In most systems pharmacology studies, the components in TCMPs were mechanically collected from chemical databases by simple summation of the compounds in each herb. As a matter of fact, the chemical substances and composition ratios of TCMPs differ dramatically due to different preparation processes. For example, tanshinones, as the main components from ethanol extract of Radix Salviae Miltiorrhizae, could not be experimentally detected in DHI because of its water extraction process [34, 35]. Hence, the chemical ingredients in DHI and NXT were collected from the high-resolution LC-MS profile data reported by related literatures to guarantee the accuracy and objectivity of this work [34–38]. Since the glycosides in NXT might be deglycosylated by the glycosidase in the intestinal tract, 14 aglycones were also considered as chemical ingredients labeled by _qt. As a result, a total of 293 ingredients were retrieved for DHI (65) and NXT (228). The detailed information of these ingredients was displayed in Table S1.

Although TCMPs usually contain tens or hundreds of chemical ingredients, only a few of them with favorable pharmacodynamic and pharmacokinetic properties are responsible for their therapeutic effects. Drug-likeness (DL) is an important drug research parameter that used to assess whether a compound functions as a drug. Drug-like compounds are those which ‘contain functional groups and/or have physical properties consistent with the majority of known drugs' [39]. Therefore, an optimal DL model based on molecules in the DrugBank database (https://www.drugbank.ca/) was employed to prescreen potential active ingredients in DHI and NXT. In this study, DL ≥ 0.18 was used to select candidate compounds for further research. Since NXT is consumed orally, oral bioavailability (OB ≥ 30%) and Caco-2 cell permeability (Caco-2 > –0.4) were also used to screen the candidate ingredients from NXT [31–33]. Further, a few compounds that do not meet these criteria but possessed high amounts and extensive pharmacological activities were also adopted. Consequently, 35 and 82 candidate ingredients were selected from DHI and NXT, respectively. Among them, 10 ingredients were common for both DHI and NXT, such as chlorogenic acid, hydroxysafflor yellow A, ferulic acid, rosmarinic acid, and salvianolic acid B (Table 1).

### 2.2. Target Collection and Analysis of TCMP-Compound-Target-Disease Network

**Figure 2:**
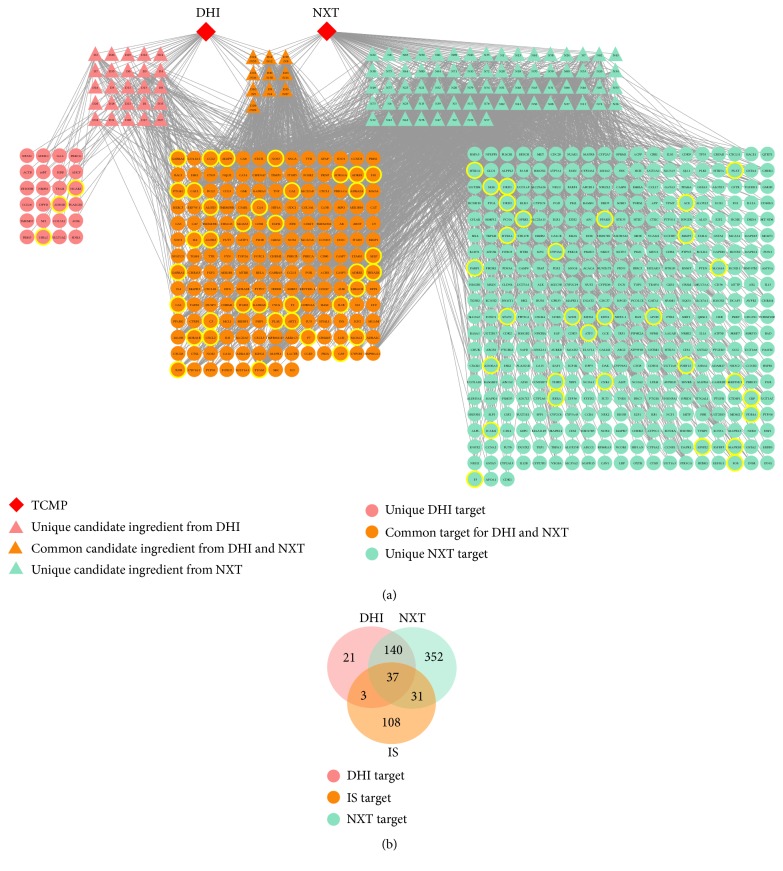
(a) TCMP-Compound-Target-Disease network. The IS targets targeted by the ingredients of DHI and NXT were labeled by yellow circle. (b) Venn diagram showing the number of common and unique targets of the candidate ingredients from DHI and NXT in treating IS.

The putative targets of the candidate ingredients in DHI and NXT were derived from Search Tool for Interactions of Chemicals and Proteins Database (STITCH, http://stitch.embl.de/), Traditional Chinese Medicine Systems Pharmacology Database and Analysis Platform (TCMSP, http://lsp.nwu.edu.cn/tcmsp.php) [40] and Binding DB (https://www.bindingdb.org/bind/index.jsp). Finally, 584 putative targets (177 targets were common for both DHI and NXT) were collected from 201 DHI-ingredient targets and 560 NXT-ingredient targets. Meanwhile, IS targets were gathered from Therapeutic Target Database (TTD, http://bidd.nus.edu.sg/group/cjttd/), DrugBank (https://www.drugbank.ca/), PharmGKB (https://www.pharmgkb.org/), MalaCards (http://www.malacards.org/), Comparative Toxicogenomics Database (CTD, http://ctdbase.org/), and then manually supplemented through a wide-scale text-mining method [41, 42]. The detailed information of all the targets was displayed in Table S2. A total of 179 targets were found to be associated with IS. Among them, 71 targets could be targeted by the candidate ingredients of DHI and NXT. Of note, 37 targets were shared by both of them; 40 and 68 targets were regarded as direct anti-IS targets of DHI and NXT, respectively (Figure 2(b)). These results showed that the targets overlapped dramatically in these two TCMPs, which also indicated different ingredients in DHI and NXT shared common or similar targets with synergistic effects.

For the purpose of interpreting the complex relationships among TCMPs, candidate ingredients, putative targets, and IS, a TCMP-Compound-Target-Disease network (Figure 2(a)) was established using Cytoscape (version 3.6.0). This network consisted of 693 nodes (2 TCMPs, 107 candidate ingredients and 584 targets) and 2561 compound-target interactions. The IS targets targeted by the ingredients of DHI and NXT were labeled by yellow circle. The degree of a node, defined the number of edges connected to it, was analyzed by Network Analysis plugin. The average degree of candidate ingredients was 25.03 and 29 ingredients possessed degrees greater than 25, indicating that these ingredients were more likely to exert therapeutic effects by acting on multiple targets. Among them, the one with a higher degree was more important in the network [43], especially quercetin (degree = 291), apigenin (degree = 143), kaempferol (degree = 128), luteolin (degree = 126), rutin (degree = 116), genistein (degree = 116), caffeic acid (degree = 108), rosmarinic acid (degree = 90), ferulic acid (degree = 76), and biochanin A (degree = 67). For example, in focal cerebral ischemia rats, quercetin can decrease cell apoptosis, prevent free radicals associated with oxidative damage, and reduce the elevated MMP-9 activity, suggesting that quercetin might have a potential role in the treatment of IS [44–46]. Rosmarinic acid, a representative compound in both DHI and NXT, showed protective effects against IS induced brain injury by synaptogenic activity and anti-inflammatory action [47, 48].

As shown in the TCMP-Compound-Target-Disease network (Figure 2(a)), the ingredients of DHI and NXT could not only modulate the crucial targets involved in the regulation of the vasomotor system and the disruption of blood-brain barrier (PTGS2, F2, ADRB1, and EGFR), but also could concentrate on the redox imbalance, neurotrophin activity, and brain inflammation (IL6, ALDH2, CCL2, STAT3, and AKT1). These processes were closely associated with IS pathogenesis. For example, increased F2 generation was associated with the risk of IS in the elderly [49, 50]. The protein F2 was found to be connected with 33 ingredients, among which danshensu, salvianolic acid C and kaempferol were reported to inhibit the activity of F2 [51–53]. In addition, the neurotoxic proinflammatory mediator PTGS2 also had interactions with multiple ingredients. Specifically, ferulic acid, tanshinone IIA, and (Z)-ligustilide could exert anti-inflammatory effects by attenuating the PTGS2 expression [54–56].

### 2.3. Comparative Study of the Anti-IS Mechanisms of DHI and NXT

**Figure 3:**
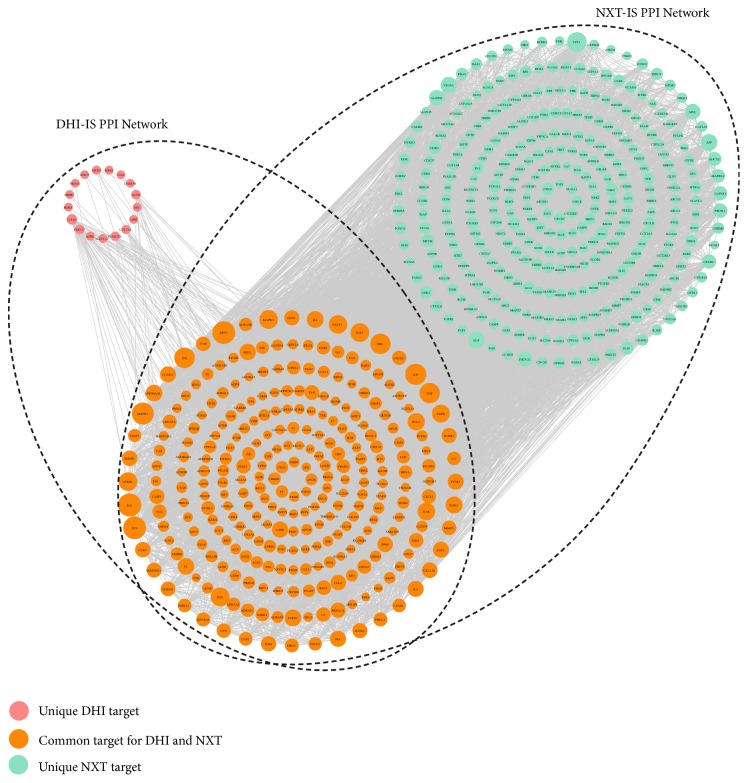
The merged DHI-IS and NXT-IS PPI network. The map node size gets larger with increased degree.

The interaction between putative targets and IS targets were derived by mapping them onto a PPI network. In this section, the IS targets and ingredient targets from DHI or NXT were integrated into STRING database (https://string-db.org/) to acquire PPI data, respectively. The species was limited to “Homo sapiens” and the confidence score was set as 0.7. Irrelevant proteins, including the putative targets and IS targets that could not directly or indirectly interact with each other, were removed. Two PPI networks, named DHI-IS and NXT-IS PPI network, were generated by Cytoscape and then merged to identify common and unique targets of DHI and NXT (Figure 3). A hub node should be two times greater than the average node degree in the network [57]; 47 and 75 hub nodes were identified for DHI-IS and NXT-IS PPI network, respectively. In the DHI-IS PPI network, 29 hub nodes were IS targets and 29 were DHI-ingredient targets. Besides the 11 overlapping targets, the remaining 18 DHI-ingredient targets were regarded as indirect anti-IS targets of DHI, illustrating the important indirect interactions with the IS targets. While in NXT-IS PPI network, 23 out of 75 hub nodes were IS targets, 67 were NXT-ingredient targets, and 15 were overlapping targets. From them, 52 targets were considered as indirect anti-IS targets of NXT.

**Figure 4:**
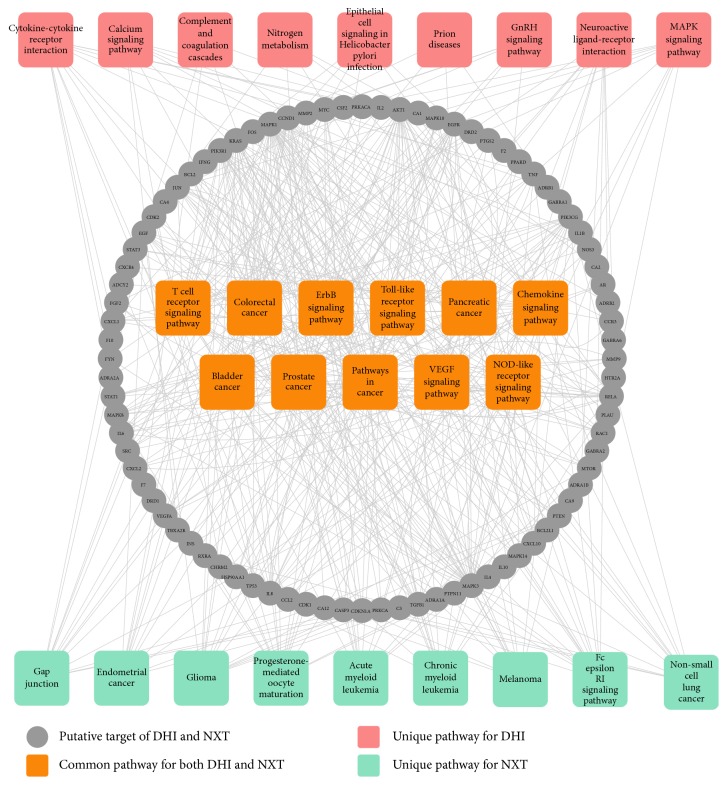
The merged T-P network for exploration of the comparative effect of DHI and NXT.

To comparatively dissect the underlying mechanisms of DHI and NXT, 58 putative targets of DHI (combining 40 direct anti-IS targets with 18 indirect anti-IS targets) and 120 putative targets of NXT (combining 68 direct anti-IS targets with 52 indirect anti-IS targets) were employed to conduct pathway enrichment analysis, respectively. The pathway information was achieved from Database for Annotation, Visualization and Integrated Discovery (DAVID, https://david.ncifcrf.gov/). The top 20 pathways (Benjamini Hochberg corrected* p* value < 0.05) for DHI and NXT were shown in Figure 4. DHI and NXT shared 11 common pathways, including 4 immune system pathways (T cell receptor signaling pathway, toll-like receptor signaling pathway, chemokine signaling pathway, and NOD-like receptor signaling pathway), 2 signal transduction pathways (ErbB signaling pathway and VEGF signaling pathway), and 5 cancer-related pathways (colorectal cancer, pancreatic cancer, bladder cancer, prostate cancer, and pathways in cancer). Among them, T cell receptor signaling pathway, toll-like receptor signaling pathway, chemokine signaling pathway, NOD-like receptor signaling pathway, and VEGF signaling pathway were closely associated with cardiocerebrovascular diseases [58]. For example, angiogenesis was observed in stroke patients, which contained extracellular matrix degradation, endothelial cell proliferation, and new vessel formation [41]. Activation of VEGF signaling pathway played a vital role in angiogenesis [59], which had also been regarded as a promising strategy for IS treatment [60]. Consistent with Wan's report [22], DHI and NXT could both activate VEGF signaling pathway and promote angiogenesis.

Besides these common pathways, DHI was also involved in 5 IS-related pathways, including cytokine-cytokine receptor interaction, calcium signaling pathway, complement and coagulation cascades, neuroactive ligand-receptor interaction, and MAPK signaling pathway. Among them, the calcium signaling pathway participated in the regulation of agonist-stimulated contraction and myogenic tone in vascular smooth muscle cells [61]. Previous study suggested that modification of calcium activity may be a novel therapeutic strategy for cardiocerebrovascular diseases, such as IS [62]. While for NXT, the 120 putative targets were also enriched in gap junction, endometrial cancer, glioma, progesterone-mediated oocyte maturation, acute myeloid leukemia, chronic myeloid leukemia, melanoma, Fc epsilon RI signaling pathway, and non-small-cell lung cancer. Besides the 6 cancer-related pathways, gap junction may play a crucial role during the development of IS due to its vital function in neuroprotection [63, 64].

Overall, it could be concluded that DHI and NXT exerted the therapeutic effects in the treatment of IS mainly through immune system pathways and VEGF signaling pathway. Furthermore, DHI could also focus on some signaling pathways, while NXT involved in gap junction. These results demonstrated that the anti-IS mechanisms of DHI and NXT had similarities and differences, which may provide important evidence for clinical choice of these two TCMPs.

### 2.4. Elucidation of the Synergistic Mechanism of DHI and NXT

**Figure 5:**
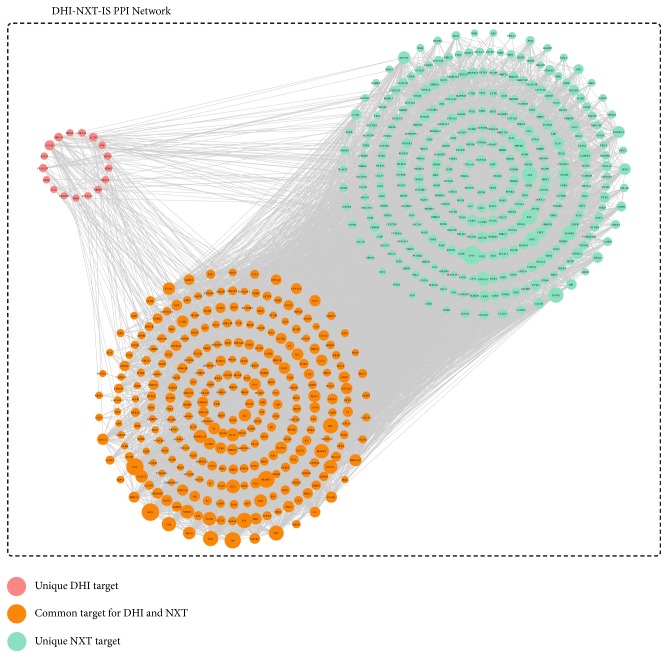
The DHI-NXT-IS PPI network. The map node size gets larger with increased degree.

To reveal the synergistic mechanism of DHI and NXT, in this section, the 179 IS targets and 584 ingredient targets (including 201 DHI-ingredient targets and 560 NXT-ingredient targets) were entirely integrated into STRING database to acquire PPI data. The DHI-NXT-IS PPI network was shown in Figure 5. The average degree of all the nodes was 23.25 and 76 nodes had degree bigger than 46. Among these hub nodes, 24 were IS targets and 68 were ingredient targets. Besides the 16 overlapping targets, the remaining 52 targets were considered as indirect anti-IS targets of this TCMP pair.

**Figure 6:**
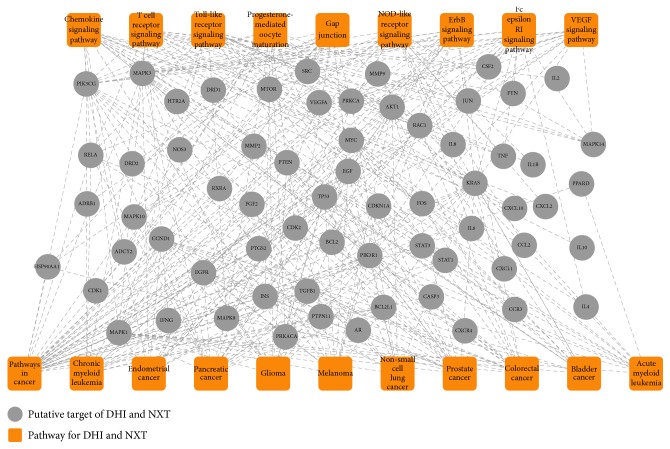
The T-P network for elucidation of the synergistic mechanism of DHI and NXT. The lower 11 pathways were associated with cancers and the upper 9 pathways were related to other processes.

The 123 putative targets of DHI and NXT (combining 71 direct anti-IS targets with 52 indirect anti-IS targets) were employed to elucidate the synergistic mechanism of DHI and NXT against IS using DAVID database. The information of top 20 pathways (Benjamini Hochberg corrected* p* value < 0.05) was summarized in Figure 6. Six pathways had high correlations with cardiocerebrovascular diseases, including four immune system pathways (chemokine signaling pathway, T cell receptor signaling pathway, toll-like receptor signaling pathway, and NOD-like receptor signaling pathway), one signaling pathways (VEGF signaling pathway), and one cell-related pathway (gap junction) [58]. Studies have reported that focal cerebral ischemia could result in the activation of inflammatory cytokines, chemokines, and chemokine receptors in the immune system. These processes may be caused by the formation of thrombi in blood vessels, particularly inside microvessels [65, 66]. Meanwhile, NOD-like receptor signaling pathway was also closely associated with the putative targets of DHI and NXT. Recent studies have reported that NOD-like receptor played an important role in central nervous diseases, such as IS. The abnormal changes of NOD-like receptors can cause redox imbalance in the brain, which had a marked impact in the pathogenesis of IS [67]. Of note, DHI and NXT showed synergistic effect in activation of the VEGF signaling pathway, as proved experimentally by Wan's study [22]. Fortuitously, 11 cancer-related pathways were also discerned in Figure 6. Danshensu from DHI was validated to exhibit antitumor activity by affecting on tumor angiogenesis and tumor cell invasion [68]. Additionally, butylidenephthalide, senkyunolide A, and (Z)-ligustilide from NXT showed selective cytotoxic and antiproliferative effects in human colon cancer cells [69].

In summary, DHI and NXT exerted the synergistic effect against IS by regulating 6 meaningful pathways, which indicated that the combination of DHI and NXT may provide better protective effect than monotherapy in immune system, angiogenesis, and neuroprotection.

## 3. Materials and Methods

### 3.1. Chemical Ingredients Collection and ADME Evaluation

The high-resolution LC-MS profile data of DHI and NXT were manually gathered from relevant researches. In order to explore the potential bioactive compounds from DHI and NXT, three important parameters were employed using* in silico* ADME models, including OB, Caco-2, and DL. Those ingredients with OB ≥30%, Caco-2 >−0.4 and DL ≥0.18 were preserved and adopted as candidate ingredients for further analysis. The details of all the ingredients and candidate ingredients were described in Tables S1 and 1, respectively.

### 3.2. Target Identification

With names and/or chemical structures (denoted by the canonical SMILES format) as key words, the putative targets of the candidate ingredients were derived from STITCH, TCMSP, and Binding DB. The obtained targets were normalized to the official gene symbols using UniProt database (https://www.uniprot.org/) with the species limited to “Homo sapiens”.

The targets related to IS were derived from five public databases, including TTD, DrugBank, PharmGKB, MalaCards, CTD with keywords “ischemic stroke”, “cerebral infarction”, “cerebral ischemia”, “brain ischemia”, and “cerebrovascular ischaemia”. Then the targets were manually supplemented through a wide-scale text-mining method and annotated using the official gene symbols as described above.

### 3.3. Network Construction and Analysis

To further obtain a global perspective of the complex relationships among compounds, targets, diseases, and pathways, several networks were constructed.

(1) TCMP-Compound-Target-Disease network. TCMPs, candidate ingredients of DHI and NXT and their putative targets, were employed to build this network. The IS targets targeted by the ingredients of DHI and NXT were labeled by yellow circle.

(2) Merged DHI-IS and NXT-IS PPI network and corresponding Target-Pathway (T-P) network. Based on the PPI data derived from STRING, with the species limited to “Homo sapiens” and a confidence score >0.7, two PPI networks named DHI-IS and NXT-IS PPI network were constructed for the ingredient targets of DHI and NXT interacting with IS targets, respectively. These two networks were merged to identify overlapping and distinct targets of DHI and NXT. Then the anti-IS targets of DHI and NXT were employed to conduct pathway enrichment analysis, respectively. The enriched pathway information of these targets was obtained from DAVID (used for pathway analysis according to Kyoto Encyclopedia of Genes and Genomes, KEGG), and then T-P networks composed of anti-IS targets of DHI or NXT and their corresponding pathways were created, respectively. These two T-P networks were merged to explore common and unique mechanisms of DHI and NXT for treating IS.

(3) DHI-NXT-IS PPI network and corresponding T-P network. A DHI-NXT-IS PPI network was established among DHI-ingredient targets, NXT-ingredient targets, and IS targets. Then the anti-IS targets were used to performed pathway enrichment analysis using DAVID database. The T-P network was generated to elucidate the synergistic mechanism of DHI and NXT in the treatment of IS.

All these networks were created by Cytoscape 3.6.0, open software for visualizing, integrating, modeling, and analyzing the biological networks.

## 4. Conclusions

To provide evidence for the better clinical use of TCMPs, comparison of the pharmacological mechanisms between TCMPs with similar therapeutic effect is necessary. DHI and NXT are representative TCMPs for IS treatment, which are also frequently used in combination. However, their independent and combined mechanisms have not been clearly clarified. In the present study, a feasible network pharmacology strategy based on chemical, pharmacokinetic, and pharmacological data was employed to elucidate the comparative and synergistic effects of DHI and NXT against IS. By systematic analysis of the constructed networks, we could learn that (1) the candidate ingredients of DHI and NXT not only could target the vasomotor system-related and blood-brain barrier-related proteins (PTGS2, F2 ADRB1, and ADRB2), but also could involve in the redox imbalance, neurotrophin activity, and brain inflammation (IL6, ALDH2, CCL2, STAT3, and AKT1). (2) DHI and NXT exerted the protective effects mainly through immune system and VEGF signaling pathways. But DHI and NXT also had their own unique pathways for treating IS, e.g., calcium signaling pathway for DHI and gap junction for NXT. (3) The synergistic mechanism of DHI and NXT was mainly associated with chemokine signaling pathway, T cell receptor signaling pathway, VEGF signaling pathway, gap junction, and so on. Overall, this study provided an effective approach for dissecting the comparative and synergistic effects of DHI and NXT in the treatment of IS.

## Figures and Tables

**Table 1:** The detailed information of candidate ingredients in DHI and NXT.

**ID**	**Source**	**Compound**	**CAS**	**OB (**%**)**	**Caco-2**	**DL**
D1	DHI	p-Hydroxybenzoic acid-O-glucoside	N/A	42.9	-1.22	0.20
D2	DHI	5-Hydroxymethylfurfural	67-47-0	45.07	0.05	0.02
D3	DHI	Danshensu	76822-21-4	36.91	-0.27	0.06
D4	DHI	Protocatechuic acid	99-50-3	25.37	0.10	0.04
D5	DHI	Neochlorogenic acid	906-33-2	18.05	-1.37	0.33
D6	DHI	Dihydrocaffeic acid	71693-95-3	32.79	0.25	0.05
D7	DHI	Protocatechuic aldehyde	139-85-5	38.35	0.43	0.03
D8	DHI	Syringin	118-34-3	14.64	-1.01	0.32
D9	DHI	Chlorogenic acid	202650-88-2	11.93	-1.03	0.33
D10	DHI	Hydroxysafflor yellow A	78281-02-4	3.53	-3.03	0.68
D11	DHI	Cryptochlorogenic acid	905-99-7	24.5	-1.43	0.33
D12	DHI	Ferulic acid	537-98-4	39.56	0.47	0.06
D13	DHI	Caffeic acid	501-16-6	25.76	0.21	0.05
D14	DHI	Roseoside	54835-70-0	10.81	-1.25	0.36
D15	DHI	Sweroside	14215-86-2	4.96	-1.08	0.38
D16	DHI	Morroniside	25406-64-8	13.86	-2.01	0.50
D17	DHI	p-Coumaric acid	7400-08-0	43.29	0.46	0.04
D18	DHI	Eriocitrin	13463-28-0	4.52	-2.23	0.7
D19	DHI	Rutin	153-18-4	3.2	-1.93	0.68
D20	DHI	Salvianolic acid I	153765-45-8	N/A	N/A	N/A
D21	DHI	Salvianolic acid J	N/A	43.38	-0.82	0.72
D22	DHI	Salviaflaside	178895-25-5	N/A	N/A	N/A
D23	DHI	Kaempferol-3-O-rutinoside	17650-84-9	3.64	-1.77	0.73
D24	DHI	Safflor yellow A	85532-77-0	22.75	-2.52	0.75
D25	DHI	Salvianolic acid D	142998-47-8	1.57	-0.78	0.50
D26	DHI	Salvianolic acid G	N/A	45.56	-0.14	0.61
D27	DHI	Salvianolic acid F	N/A	N/A	N/A	N/A
D28	DHI	Salvianolic acid E	N/A	3.01	-1.52	0.39
D29	DHI	Rosmarinic acid	20283-92-5	1.38	-0.54	0.35
D30	DHI	Lithospermic acid	28831-65-4	2.67	-1.07	0.76
D31	DHI	9′′-Methyl lithospermate B	N/A	3.01	-1.01	0.39
D32	DHI	Salvianolic acid B	115939-25-8	3.01	-1.67	0.41
D33	DHI	Salvianolic acid L	N/A	N/A	N/A	N/A
D34	DHI	Salvianolic acid A	96574-01-5	2.96	-0.56	0.7
D35	DHI	Salvianolic acid C	115841-09-3	2.5	-0.23	0.81
N1	NXT	Gallic acid	149-91-7	31.69	-0.09	0.04
N2	NXT	Mulberroside A	166734-06-1	13.34	-2.43	0.73
N3	NXT	Catechin	154-23-4	54.83	-0.03	0.24
N4	NXT	Chlorogenic acid	202650-88-2	11.93	-1.03	0.33
N5	NXT	Hydroxysafflor yellow A	78281-02-4	3.53	-3.03	0.68
N6	NXT	Vanillic acid	121-34-6	35.47	0.43	0.04
N7	NXT	Epicatechin	35323-91-2	48.96	0.02	0.24
N8	NXT	Albiflorin	39011-90-0	12.09	-1.54	0.77
N9	NXT	Rutin	153-18-4	3.2	-1.93	0.68
N10	NXT	Paeoniflorin	23180-57-6	53.87	-1.47	0.79
N11	NXT	Paeoniflorin_qt	23180-57-6	68.18	-0.34	0.40
N12	NXT	Ferulic acid	537-98-4	39.56	0.47	0.06
N13	NXT	Calycosin-7-O-glucoside	20633-67-4	5.49	-1.13	0.81
N14	NXT	Kaempferol-3-O-rutinoside	17650-84-9	3.64	-1.77	0.73
N15	NXT	Biochanin A	491-80-5	25.21	0.65	0.24
N16	NXT	Salvianolic acid B	115939-25-8	3.01	-1.67	0.41
N17	NXT	Ononin	486-62-4	11.52	-0.74	0.78
N18	NXT	Calycosin	20575-57-9	47.75	0.52	0.24
N19	NXT	Formononetin	485-72-3	69.67	0.78	0.21
N20	NXT	Senkyunolide A	63038-10-8	26.56	1.30	0.07
N21	NXT	Tanshinone II-B	17397-93-2	65.26	0.44	0.45
N22	NXT	Z-Butylidenephthalide	551-08-6	42.44	1.32	0.07
N23	NXT	(E)-ligustilide	4431-01-0	51.3	1.30	0.07
N24	NXT	(Z)-ligustilide	4431-01-0	23.5	1.28	0.07
N25	NXT	Cryptotanshinone	35825-57-1	52.34	0.95	0.40
N26	NXT	Tanshinone IIA	568-72-9	49.89	1.05	0.40
N27	NXT	Caffeic acid	501-16-6	25.76	0.21	0.05
N28	NXT	Amygdalin	29883-15-6	4.42	-1.91	0.61
N29	NXT	Rosmarinic acid	20283-92-5	1.38	-0.54	0.35
N30	NXT	Lithospermic acid	28831-65-4	2.67	-1.07	0.76
N31	NXT	Wogonin	632-85-9	30.68	0.79	0.23
N32	NXT	Senkyunolide H	94596-27-7	34.34	-0.02	0.10
N33	NXT	Neocnidilide	4567-33-3	83.83	1.23	0.07
N34	NXT	Mulberrin	19275-47-9	1.22	0.46	0.59
N35	NXT	Sugiol	511-05-7	36.11	1.14	0.28
N36	NXT	Lindestrene	2221-88-7	36.12	1.75	0.13
N37	NXT	(z)-9-Octadecenamide	301-02-0	31.2	1.26	0.14
N38	NXT	Senkyunolide B	93236-67-0	62.68	1.00	0.08
N39	NXT	Senkyunolide C	91652-78-7	46.8	0.87	0.08
N40	NXT	7-Hydroxycoumarin	93-35-6	27.37	0.74	0.05
N41	NXT	Pratensein	2284-31-3	39.06	0.39	0.28
N42	NXT	Astragaloside IV	83207-58-3	17.74	-2.22	0.15
N43	NXT	Astragaloside II	84676-89-1	46.06	-2.05	0.13
N44	NXT	Methyl tanshinonate	N/A	19.19	0.56	0.55
N45	NXT	Hydroxytanshinone IIA	18887-18-8	44.93	0.53	0.44
N46	NXT	Kumatakenin	3301-49-3	50.83	0.61	0.29
N47	NXT	Trijuganone B	126979-84-8	38.75	0.96	0.36
N48	NXT	Carthamidin	479-54-9	41.15	0.16	0.24
N49	NXT	Acetyl-11-keto-*β*-boswellic acid	67416-61-9	17.79	0.20	0.67
N50	NXT	Scopolin	531-44-2	25.5	-0.88	0.39
N51	NXT	Daidzoside	552-66-9	14.32	-1.00	0.73
N52	NXT	Ecdysterone	5289-74-7	5.3	-1.32	0.82
N53	NXT	Ethyl gallate	831-61-8	25.61	0.33	0.06
N54	NXT	Luteolin	491-70-3	36.16	0.19	0.25
N55	NXT	Safflor yellow A	85532-77-0	22.75	-2.52	0.75
N56	NXT	Scopoletin	92-61-5	27.77	0.71	0.08
N57	NXT	Luteolin-7-O-*β*-D-glucopyranoside	26811-41-6	7.29	-1.23	0.78
N58	NXT	Oxyresveratrol	4721/7/7	109.29	0.55	0.13
N59	NXT	Tanshindiol C	96839-30-4	42.85	-0.04	0.45
N60	NXT	Quercetin	117-39-5	46.43	0.05	0.28
N61	NXT	Tanshindiol B	97465-70-8	42.67	0.05	0.45
N62	NXT	Aloe emodin	481-72-1	83.38	-0.12	0.24
N63	NXT	Genistein	446-72-0	17.93	0.43	0.21
N64	NXT	Kaempferol	520-18-3	41.88	0.26	0.24
N65	NXT	Palbinone	139954-00-0	43.56	0.00	0.53
N66	NXT	Farrerol	24211-30-1	42.65	0.59	0.26
N67	NXT	Astragaloside I	84680-75-1	46.79	-2.28	0.11
N68	NXT	Danshenxinkun A	65907-75-7	45.64	0.48	0.30
N69	NXT	E-butylidenephthalide	551-08-6	53.72	1.30	0.07
N70	NXT	Dihydrotanshinone I	87205-99-0	45.04	0.95	0.36
N71	NXT	Furanodiene	19912-61-9	45.11	1.77	0.10
N72	NXT	Tanshinone I	568-73-0	29.27	1.05	0.36
N73	NXT	Cyclomulberrin	19275-51-5	9.84	0.72	0.84
N74	NXT	Miltirone	27210-57-7	38.76	1.23	0.25
N75	NXT	1,2-Dihydrotanshinone	77769-21-2	19.95	1.07	0.36
N76	NXT	Methyl gallate	99-24-1	30.91	0.26	0.05
N77	NXT	Taxifolin	480-18-2	57.84	-0.23	0.27
N78	NXT	Morin	480-16-0	46.23	0.00	0.27
N79	NXT	Apigenin	520-36-5	23.06	0.43	0.21
N80	NXT	Coniferyl ferulate	63644-62-2	4.54	0.71	0.39
N81	NXT	Senkyunolide E	94530-83-3	34.4	0.55	0.08
N82	NXT	Mulberrofuran A	68978-04-1	4.45	1.04	0.53

## Data Availability

The data of our research can be acquired from the Supplementary Materials uploaded with this article.

## Supplementary Materials

Supplementary Materials Table S1: the detailed information of all compounds in DHI and NXT. Table S2: the detailed information of DHI-ingredient targets, NXT-ingredient targets, and IS targets.
